# Do Existential Variables Mediate Between Religious-Spiritual Facets of Functionality and Psychological Wellbeing

**DOI:** 10.1007/s10943-012-9597-6

**Published:** 2012-03-24

**Authors:** Marcin Wnuk, Jerzy Tadeusz Marcinkowski

**Affiliations:** 1 Department of Social Medicine, Poznań University of Medical Sciences, Rokietnicka Street 5C, 60-806 Poznan, Poland; 2 Mateckiego Street 24/71, 60-689 Poznan, Poland

**Keywords:** Religiosity, Spirituality, Hope, Meaning of life, Mediator variable, Wellbeing

## Abstract

Religiosity has been related to psychological wellbeing outcomes. Although this relationship is primarily based on studies of church attendance or prayer and wellbeing, more recent work has focused on the potential mechanisms that may mediate the religion-wellbeing findings. One of the major function of religion is finding of meaning of life and improving hope. Recent studies have indicated that hope and meaning of life are the potential variables mediate between religion and wellbeing. It was hypothesized that one pathway through which religiosity may exert its positive influence on psychological wellbeing is through finding meaning of life and improving hope. One study was conducted examining the relationships among spiritual experiences, hope, meaning of life and psychological wellbeing operationalized as satisfaction with life, positive affect and negative affect. The following research tools were used: Daily Spiritual Experiences Scale, Purpose in Life Test, Hearth Hope Index, Cantril Ladder, Positive and Negative Affect Schedule. Meaning of life and hope were noticed to mediate between spiritual experiences and satisfaction with life as well as between spiritual experiences and positive affect. Spiritual experiences were not related to negative affect. Both meaning of life and hope predicted negative affect. This study found meaning of life and hope to be an important factors in the religion-wellbeing relationship and related to positive psychological outcomes, including improved satisfaction with life and positive affect as well as reduced negative affect.

## Introduction

The survey of the literature has revealed the researchers’ trends for searching for the variables that mediate between the religious and spiritual aspects of the functionality of an individual and his or her wellbeing (Jones 2004; Park 2007; Zullig et al. 2006). The researchers have tried to confirm that faith and religious-spiritual commitment favors hope (Wnuk 2009, 2010; Wnuk et al. 2009), optimism (Park 2007; Wnuk et al. 2009; Steger and Frazier 2005), high self-assessment (Steger and Frazier 2005), finding the meaning of life (Jones 2004; Park 2007; Wnuk 2009, 2010; Wnuk et al. 2009; Steger and Frazier 2005; Zika and Chamberlain 1988; Vilchensky and Kravetz 2005; French and Joseph 1999), social support of an individual (Jones 2004; Vilchensky and Kravetz 2005), a sense of inner control (Fiori et al. 2006), subjective sense of health (Zullig et al. 2006), and employing religious coping (Roesch and Ano 2003; Schaefer and Gorsuch 1991; Nooney and Woodrum 2002; Fabricatore et al. 2004) which, in consequence, lead to an increase in individual wellbeing.

Thus far, the strongest empirical foundation has been formed by the conviction that religious-spiritual aspects of functionality are the source of the meaning of life, resulting indirectly in the improvement of psychological wellbeing. (Wnuk 2009, 2010; Wnuk et al. 2009; Steger and Frazier 2005; Zika and Chamberlain 1988; Vilchensky and Kravetz 2005; French and Joseph 1999). In the study by Steger and Frazier, conducted among the students of psychology, the meaning of life mediated between religious involvement and satisfaction with life (Steger and Frazier 2005). Another study, performed among Israeli students, revealed a mediating role of the meaning of life between religious faith and psychological wellbeing or distress (Vilchensky and Kravetz 2005).

Among the Al-Anon group members, spiritual experiences were the source of meaning of life leading indirectly to an increase in satisfaction with life as well as to the distress level decrease (Wnuk et al. 2009). A parallel study was conducted among the Alcoholics Anonymous with the application of different wellbeing indicators. The increase in the frequency of spiritual experiences was revealed to be conducive to finding the meaning of life in alcohol addicts being members of the Alcoholics Anonymous community and finally reduced the hopelessness level (Wnuk 2009).

Other studies proved that the relationship between the attitude toward Christianity and the sense of happiness was mediated through the meaning of life (French and Joseph 1999). The results of studies conducted on a large sample of women were ambiguous. Apart from one measure of the meaning of life, this variable mediated between religiousness and the cognitive aspect of wellbeing in the form of the satisfaction with life. The meaning of life turned out not to mediate between religiousness and emotional indicators of psychological wellbeing in the form of negative and positive affect, irrespective of which research tool had been employed (Zika and Chamberlain 1988). Similarly, among the Alcoholics Anonymous, the meaning of life was a variable that mediated between the religiousness indicators such as intrinsic religiousness, strength of religious faith, positive religious coping and the satisfaction with life (Wnuk 2010). That regularity has not been confirmed in relation to affective measures of psychological wellbeing, which seem to indicate that religiousness through the function of finding the meaning of life can influence only the cognitive components of wellbeing.

Among the entire range of approaches dealing with the meaning of life in psychology, the best known and widespread is logotheory, posed by the Viennese psychiatrist and philosopher, Victor Frankl (Auhagen 2000). Frankl’s inspiration to articulate this therapy originates from the time spent in a concentration camp during the World War II. He noticed that in spite of severe physical deprivation, the prisoners who were able to find the meaning in the events happening to them were able to stay alive, while those who were not able to find purpose, experienced feelings of hopelessness, emptiness or senselessness, which, coupled with loss of physical vitality and stamina, led to disease and death (Frankl 1962). According to Frankl, people have a natural disposition toward developing the meaning of life, which motivates them to analyze and search for a purpose in the events in which they participate. The later research confirmed that the ability to identify a purpose is an element *sine qua non* of the sense of happiness (Frankl 1977). Individuals having problems with finding a purpose in their lives experience a state of “existential emptiness” characterized by feeling of boredom, anhedonia, hopelessness and the lack of motivation for engaging themselves in activity (Frankl 1973).

Psychological wellbeing is a multidimensional idea. Its cognitive aspect is satisfaction with life, while its emotional indicators are positive and negative affects (Diener 1984).

Apart from the meaning of life, a religious-spiritual area of functionality seems to be an additional source of hope (Gibson 2003; Craig et al. 1999; Frey et al. 2005). Among chronically ill inhabitants of rural areas, a moderately strong positive correlation has been observed between spirituality and hope (Craig et al. 1999). In HIV-infected individuals, hope correlated positively with spiritual wellbeing (Abdel-Khalek 2006), whereas in women with breast cancer, hope correlated with the individual awareness of inner ego and the sense of connection with the “Higher Power” (Gibson 2003).

Among students, hope positively correlated with life satisfaction (Abdel-Khalek 2006; Chang 1988; Gilman et al. 2006) and positive affect (Snyder et al. 1998; Michael and Snyder 2005). The results of previous studies appear to confirm the fact that the increase in hope induced by religious-spiritual factors is manifested by better quality of life and wellbeing. Among the Alcoholics Anonymous, spiritual experiences mediated by hope were reflected by a lower level of hopelessness measured with the Beck’s Hopelessness Scale (Wnuk 2009). The results of the research by Wnuk on the same population showed that hope played a mediating role between frequency of prayer and positive affect (Wnuk 2010). Also, among the group-supported addicts, spiritual experiences, coupled with a high level of hope, indirectly influenced satisfaction with life and a distress level (Wnuk et al. 2009).

Hope has been found to be an important and valuable factor in the life of an individual. Regardless of the approach, the studies on hope highlighted its multidimensional character. Hope reflects spheres of thought, feelings, behaviors, and relationships with other people. It consists of an element of predictability; it is future oriented but rooted in the present (Stephenson 1991), and connected with the past sense of hope.

In medicine, the most frequent tool used to study hope is the Herth Hope Index. Hope is operationalized as a multidimensional, dynamic force of life, described as a certainty of achieving good, which for a hopeful individual is possible and personally important (Dufault and Martocchio 1985). According to this theory, hope is based on three main factors: the inner sense of time and future, the inner positive readiness and expectation, and the sense of connection with oneself and others. The first of them is manifested through the presence of goals, positive attitude toward the world, a conviction that every day brings a new chance, and carefree thoughts about the future. The inner positive readiness and expectation is realized through the ability to notice positive aspects, the feeling of being guided, conviction that life has meaning, and the ability to recall happy and joyful moments.

The feeling of connection with oneself and others refers both to oneself as well as to God and other people. It is realized due to the conviction of not being alone, comfort-giving faith, deep inner strength, and the ability of giving and receiving love (Herth 1992). The main hope-supporting strategies are meaningful relations with other people, the ability of carefree feeling, determination, possessing courage and serenity, clear goals, spiritual convictions (faith), the ability of recalling positive memories and respecting and accepting the individuality of others (Herth 1990).

This paper purposely integrates the religious and spiritual spheres. The terms ‘spirituality’ and ‘religiousness’ are usually used interchangeably, as equivalents. According to the previous studies, some aspects of spirituality and religiousness are closely connected, whereas other spiritual spheres are loosely connected or independent of religious behaviors and motives (Heintz and Baruss 2001). Nevertheless, spirituality seems to be an idea having a broader meaning than religiousness. Unlike religiousness, spirituality assumes the realization of non-religious goals, such as identity, affiliation, health, or wellbeing (Sawatzky et al. 2005). It means that one can develop spiritually without being religious. On the other hand, spiritual experience is the crucial element of religious development (Wnuk 2008).

The aim of the present work is to test the following hypotheses:

- The meaning of life mediates between spiritual experiences and satisfaction with life.
- The meaning of life is directly correlated with both positive and negative affect. However, spiritual experiences do not correlate with emotional indicators of psychological wellbeing in the form of positive and negative affect.
- Hope mediates between spiritual experiences and psychological wellbeing.

## Method

### Participants

The study included 115 students of the University School of Physical Education in Poznań and the Warsaw School of Social Psychology. All the students gave the consent to participate in a questionnaire study of psychosocial wellbeing and religiosity. Since the packet took approximately 1 h to complete, the students filled it at home and returned it the following class period.

### Research Tools

The following research tools were used: the Daily Spiritual Experiences Scale (DSES), the Purpose in Life Test (PIL), the Hearth Hope Index (HHI), the Cantril Ladder, and the Positive and Negative Affect Schedule (PANAS).

### Demographics

Mean age of the subjects was 22 years (*M* = 22; SD = 2.79). Of them, 86.1 % had secondary education, and 13.9 % had higher education. Women comprised 60.9 % of the study group and men—39.1 %. All the subjects declared to be Roman-Catholics.

### Independent Variables

#### Spiritual Experiences

The Daily Spiritual Experience Scale (DSES) consists of 16 questions. The respondents give answers on a 6-grade scale from 1—‘never or almost never’ to 6—‘many times during the day’. The respondents’ level of spirituality was assessed as directly proportional to the points scored by them. This tool presents satisfactory psychometric properties. Its reliability, according to the population, is placed between *α* = 0.86–0.95 (Loustalot et al. 2006). This project recorded reliability of this scale as *α* = 0.94.

##### Hope

The Herth Hope Index (HHI) is a scale used for the measurement of hope based on a definition of hope stating that it is a multidimensional, dynamic life force, which can be characterized as the certainty of achieving a good result, which for an individual with hope is possible and personally significant (Dufault and Martocchio 1985). The participants answered 12 questions expressed on the four-step Likert scale (Jones 2004; Park 2007; Zullig et al. 2006; Wnuk 2009) where total agreement with three and five number of items means 1, and total disagreement means 4. Total agreement with the rest of the items of the questionnaire means 4, and total disagreement means 1 (Herth 1992). The reliability of this scale has satisfactory psychometric features—in reference to patients scores *α* = 0. 97 (Herth 1992) were evaluated with the test–retest method scores 0.91 (Herth 1992) and in the previous research scores *α*-Cronbach = 0.91. The reliability of this tool in the described project was *α* = 0.74.

##### Meaning of life

The Purpose in Life Scale Test (PIL) consists of twenty statements concerning the need for the meaning of life. Each question is answered by marking the fields placed on the continuum between 1 and 7, with 7 indicating maximum intensity related to the meaning of life, and 1 indicating minimum intensity. The overall result is obtained by adding the score from all the answers. The higher score indicates the stronger meaning of life, the lower—the greater existential frustration. The score ranges between 20 and 140 points (Cekiera 1985). The reliability of this tool measured by the coefficient of correlation (r-Pearson) was 0.82 with the Spearman–Brown correction—0.90 (Crumbaugh and Maholic 1964). For the Polish version of the scale, when we used a test–retest method during the 6-month interval, the reliability ranged from 0.64 to 0.70, depending on the population studied (Siek 1993). The reliability of this tool in the described project was *α* = 0.91.

### Dependent Variable

Psychological wellbeing was operationalized as a global cognitive assessment of one’s own life from the perspective of satisfaction with life and the amount of positive and negative emotional states experienced by the respondents during the weekend preceding the study.

#### Satisfaction with Life

The Cantril Ladder consists of one question in which the respondents evaluate their overall satisfaction with life on a scale from 0 to 10. We used the “Onion” program combined with the Czapiński’s scale during a 2-month interval, obtaining a reliability score of 0.76 (Czapiński 1992). In another project, the coefficient of reliability in a 2-year interval was 0.65 (Kivett and Palmore 1977).

#### Positive and Negative Affect

The Positive and Negative Affect Schedule (PANAS) consists of 10 statements related to positive affect and of 10 statements related to negative affect. The responses were evaluated on a 1–5 scale; 1—a little or not at all; 5—very often. In the study, the respondents assessed their affect in relation to the weekend preceding the study. The scale reliability varied, depending on the research project, from *α* = 0.86 to *α* = 0.89 in the positive affect part, and from *α* = 0.84 to *α* = 0.85 in the negative affect part (Crawford and Henry 2004; Trawka and Derbis 2006). The reliability of this tool assessed with the test–retest method among the students was 0.39–0.71 (Trawka and Derbis 2006). In the study described in this paper, the reliability measured with the Cronbach alpha was *α* = 0.85 and *α* = 0.87 for positive and negative affect, respectively.

## Results

The values of the paired correlation coefficients are presented in Table 1. The table shows the presence of moderate, positive relationship between satisfaction with life, positive affect, hope, the meaning of life and spiritual experiences. There is a weak negative correlation between satisfaction with life and negative affect. The negative affect is negatively correlated with hope and the meaning of life, positive interrelationships between positive.

**Table 1:** Correlation matrix (*n* = *115*)

	1	2	3	4	5
1. Satisfaction with life					
2. Positive affect	0.26**				
3. Negative affect	−0.37**	−0.13			
4. Hope	0.38**	0.48**	−0.32**		
5. Meaning of life	0.51**	0.53**	−0.30**	0.65**	
6. Spiritual experiences	0.31**	0.36**	−0.02	0.49**	0.32**

* *p* ≤ 0.05

** *p* ≤ 0.01

**Fig. 1:**
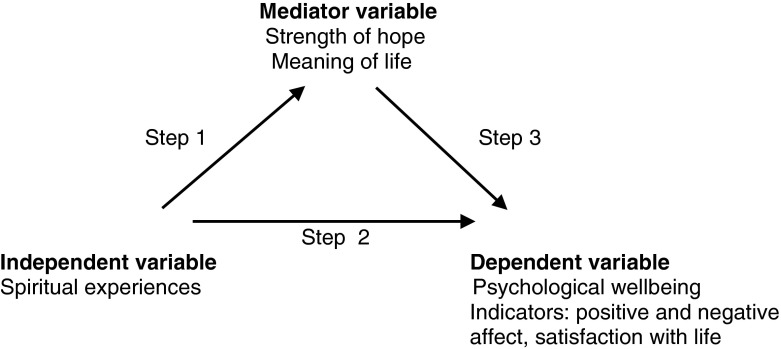
Model applying mediator variable, dependent and independent variables. *Source*: author’s analysis

A given variable plays a mediating role under specific conditions. It has to be related to a dependent and an independent variable. Simultaneously, a dependent variable must be related to an independent variable. The introduction of the three variables to the regression equation should create the situation when the mediator variable remains the predictor of the dependent variable, whereas the previously calculated statistically significant correlation between the dependent and independent variable should be reduced to a statistically insignificant level (Baron and Kenny 1986) (Fig. 1).

Further analyses were carried out with the use of multiple regression analysis. For the interrelations between the variables which did not meet the first two assumptions, no further calculations were made. Finally, 4 analyses were completed. Satisfaction with life and positive affect were used as potential mediator variables in all of them; also in the regression equation, spiritual experiences were introduced as an independent variable.

When the meaning of life was introduced to the regression equation, the relation between spiritual experiences and satisfaction with life was reduced from the statistically significant level, obtained at the earlier stage of the analysis (see Table 1), to a statistically insignificant level (β = 0.161; *p* = 0.068), whereas the meaning of life continued to be a predictor of satisfaction with life (β = 0.509; *F* = 37.46; *p* < 0.01).

When instead of satisfaction with life, positive affect was used as a dependent variable, and the correlation coefficient value of that variable and of spiritual experiences (see Table 1) was reduced to a statistically insignificant level (β = 0.211; *p* = 0.012).

Hope also turned out to be a variable mediating between spiritual experiences vs. satisfaction with life and positive affect. During the regression analysis, the strength of the relation between spiritual experiences and positive affect (see Table 1) was reduced to a statistically insignificant level (β = 0.166; *p* = 0.103) and (β = 0.161; *p* = 0.090), respectively. Simultaneously, hope remained the predictor of satisfaction with life (β = 0.383; *F* = 18.71; *p* < 0.01) and of positive affect (β = 0.482; *F* = 34.19; *p* < 0.01).

Additionally, the Sobel test was applied (Bryan et al. 2007) to confirm the mediating role of hope and the meaning of life between spiritual experiences and satisfaction with life, as well as between spiritual experiences and positive affect. It has proved that the meaning of life mediated between spiritual experiences and satisfaction with life (*z* = 3.01; *p* < 0.05), as well as between spiritual experiences and positive affect (*z* = 3.82; *p* < 0.05). It has also confirmed the mediating role of hope between spiritual experiences and satisfaction with life (*z* = 8.20; *p* < 0.05), as well as between spiritual experiences and positive affect (*z* = 2.60; *p* < 0.05).

## Discussion

The first hypothesis has been confirmed to quite a large extent. According to the results of the previous research, spiritual experiences were indirectly related to satisfaction with life, and the variable mediating the relation between them was the meaning of life (Wnuk 2010; Wnuk et al. 2009; Steger and Frazier 2005; Zika and Chamberlain 1988).

The results of the present study have confirmed a significant role of the meaning of life as a variable mediating between religious and spiritual indicators of functionality and quality of life, regardless which tools and measures were applied to verify both these variables and how different the studied populations were. It confirms the universal character of the presented model, proving that the function of the religious-spiritual sphere of existence is the discovery of the meaning of life due to which an individual can experience wellbeing, happiness, and satisfaction with life.

The aforementioned correlations have been observed in the population of students, representatives of the Alcoholics Anonymous, and women, when both positive and negative measures of wellbeing were applied such as happiness, satisfaction with life, distress, or hopelessness. There were also numerous religious-spiritual indicators employed, comprising religious involvement through the strength of faith, religious motivation, attitude toward Christianity, positive religious coping, and spiritual experiences (Wnuk 2009, 2010; Wnuk et al. 2009; Steger and Frazier 2005; Vilchensky and Kravetz 2005; French and Joseph 1999). The only exception to the above-mentioned rule was the results of the study conducted among women, with the application of affective indicators of wellbeing. This study has shown that the activity of the religious-spiritual sphere was neither directly nor indirectly related to quality of life (Zika and Chamberlain 1988).

Contrary to the previous study, the meaning of life mediated between spiritual experiences and one of the two emotional indicators of wellbeing in the form of positive affect (Zika and Chamberlain 1988). These differences can be explained with the dissimilarities between the studied populations and different measures applied to investigate the religious-spiritual sphere. This means that the students’ spiritual experiences have the influence, through the mediating role of the meaning of life, not only on the cognitive indicators of psychological wellbeing, but also on its emotional aspects, however, only on those of a positive nature.

The results of this study have revealed that one of the crucial functions of religiousness/spirituality was the discovery of the meaning of life, which could be explained on the basis of the mechanisms of coping with stress by means of religious-spiritual values. The religious-spiritual activity through the meaning can play an important role in the process of coping with stress, in the stage of both primary and secondary assessment of the stress-generating situation. In the stage of the primary assessment, the influence of a stressor can be restricted due to finding the meaning of the events through belief that no evil can happen without God’s allowance, or through the perception of the situation by an individual as a test of his or her faith. In the secondary stage of the assessment, an individual can reduce the negative influence of a stressful situation by discovering its meaning through acceptance, religious reattribution, due to comprehension why a specific event happened, or due to a positive reinterpretation through identification and focusing on positive implications of the event (Park 2005).

The concepts of spirituality highlight four main dimensions such as relation toward the “Higher Power”, own self, other people, and natural environment (Tse et al. 2005; Martsolf and Mickley 1998; Bloch 2004; Ingersoll and Bauer 2004; Dyson et al. 1997). According to them, the crucial element, around which spiritual functionality of individuals concentrates, is the meaning of life (Dyson et al. 1997; Chiu et al. 2004; Graham et al. 2001; Allen and Coy 2004; Genia 2001). The observed positive correlation between spiritual experiences and the meaning of life seems to prove this assumption.

A frequent feeling of coalescence with God, self-trust, a sense of integrity, identity and fellowship with others, as well as the awareness of the support of God and other people, probably allow the students to overcome their mental, biological, and social barriers. Due to it, they are able to find the meaning in their lives.

A moderate nature of the relationship between the meaning of life and all applied indicators of psychological wellbeing shows a significant role of that variable in the students’ quality of life. The methods used in this study to a considerable extent support Frankl’s observations (Frankl 1977), which indicated that finding the meaning of life leads to happiness. Happiness is the result of an effective discovery of the meaning in various situations and events in the life of an individual.

The second hypothesis has been partially confirmed by our analyses. Similarly to the meaning of life, it was hope, which mediated between spiritual experiences and satisfaction with life, as well as between spiritual experiences and positive affect. The obtained results are concurrent with the results of studies among addicted individuals, where identical measures of wellbeing and religiousness/spirituality were used (Wnuk 2010). Also, the use of other indicators of wellbeing among the members of the Alcoholics Anonymous, the meaning of life turned out to be a mediating factor between spiritual experiences and the sense of hopelessness (Wnuk 2009).

Our study has confirmed a significant role of religious-spiritual activity in creation of hope. It is highly probable that hope is positively influenced by the spiritual experiences manifested by the feeling of the presence of God, being the main source of joy, strength, peace and balance, feeling of God’s help and guidance, experiencing God’s love directly or via other persons, acceptance of other people regardless of their activities, feeling of integrity with own life, of identity with the beauty of the surrounding world, and selfless care for others. Hope, in turn, is reflected in positive goals, positive perception of the world, conviction that every day is a new chance, confidence in good future, positive thinking, feeling of guidance, appreciating the value of life, ability of recalling happy memories, belief in not being alone, faith which gives comfort, inner strength, and ability to give and receive love. The enumerated above factors decide on the students’ wellbeing, which is indicated by satisfaction with life and positive affect revealed by such emotions as excitement, inspiration, pride, positive attitude, determination, etc.

The previous studies showed that hope was the predictor of satisfaction with life (Abdel-Khalek 2006; Chang 1988; Gilman et al. 2006) and positive affect among the students, which confirms its crucial role in the quality of life. Another finding was that the negative correlation between the strength of hope and negative affect.

Our studies have confirmed a positive role of hope and the meaning of life in creating students’ mental wellbeing. Identified relations concerned all indicators of mental wellbeing.

According to Park’s model, both the meaning of life and hope were found to be variables mediating the relation between religiousness/spirituality and positive indicators of psychological wellbeing in form of satisfaction with life and positive affect (Park 2007). Although the study was conducted on a transverse model, the applied methods allow to determine, with high probability, the direction of relations between the used variables (Baron and Kenny 1986). Frequent spiritual experiences promote hope and the meaning of life, the consequences of which are more frequently occurring positive emotions and satisfaction with students’ life.

Longitudinal studies could validate more the obtained relations between the above-mentioned variables by presenting them in the cause-effect perspective.

Designing a similar study among individuals experiencing a difficult situation in life such as among persons chronically ill, with the application of a greater number of religious-spiritual indicators, and a greater number and variety of measures of quality of life could give more interesting outcomes. The discovery of other sources of hope and the meaning of life, apart from religious-spiritual ones, might be employed in the designing of therapeutic activities for various groups of patients, especially for those who regard religious-spiritual values as insignificant or totally unimportant.

The inclusion of more differentiated study groups in terms of socio-demographic variables would allow to find out whether these variables are important for the relationship between religious-spiritual aspects of existence and quality of life. Such variables could modify the relations between religiousness/spirituality and quality of life. In the described project, nearly all subjects were peers, with similar education and affiliation to the same religious society, which enabled the testing of the influence of these variables.

## Conclusions

The meaning of life and hope were related to both cognitive and emotional measures of psychological wellbeing, and mediated the majority of the relationships between spiritual experiences and quality of life. This is not the first study which examines the meaning of life and hope as potential pathways through which religiosity/spirituality influences quality of life and wellbeing. In the present study, this mechanism was confirmed once again with the use of specific measures of religiosity/spirituality, wellbeing and students’ population.
